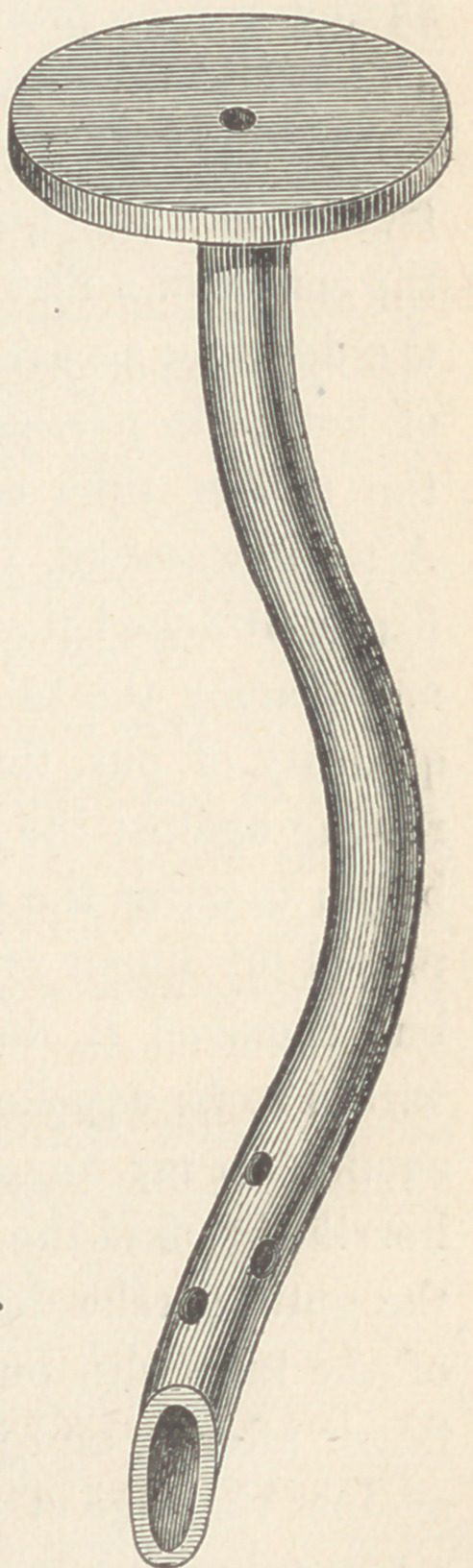# A Simple Suction Drainage Tube for Suppurating Pleural Cavities

**Published:** 1882-11

**Authors:** Edmund Andrews

**Affiliations:** Professor of Clinical Surgery in Chicago Medical College; No. 6 Sixteenth St., Chicago


### ARTICLE III.

A SIMPLE SUCTION DRAINAGE TUBE FOR SUPPURATING PLEURAL CAVITIES. By EDMUND ANDREWS, M.D., LL.D., Professor of Clinical Surgery in Chicago Medical College.

Air admitted to a suppurating pleural cavity is perfectly innocent, if accompanied by a proper carbolated injection. This truth, which I am prepared to sustain by numerous cases in practice, dispenses at once with all the laborious plans recommended in our authorities for the purpose of excluding the atmosphere from suppurating pleuras.

In saying that air thus carbolated is innocent, I mean only that it produces no objectionable septic or chemical effects. It is desirable, however, that a moderate portion of it should be withdrawn, so as to keep up a partial vacuum in the thorax, whose suction shall tend to hasten the expansion of the lung and bring it sooner into contact with the walls of the chest, thus shortening the treatment and preventing the ordinary contraction of the thoracic walls.

An ingenious, but clumsy plan was devised for this purpose by Goodhart, of Guy's Hospital, London (*Guy's Hospital Report*, 1877). He inserts a long rubber tube into the chest, and lays the outer end in a vase of water set upon the floor beside the patient's bed. The column of pus thus running downward, acts by its weight to produce a slight suction in the pleural cavity, and thus tends, to a trifling extent, to expand the lung. The great objection to the plan is that the patient cannot walk about, nor even turn freely in bed. Dr. Goodhart found it to drain but imperfectly in nearly two-thirds of the cases. Bryant condemns the plan, and most surgeons who use the pleural tubes at all, give up the attempt to produce a vacuum. An ingenious plan of a tube with valves and other attachments was published by another author, but seems to have fallen out of notice, probably on account of its complexity.

M. Chassaignac, so often quoted in this connection, made no effort to secure a vacuum, but only tried clumsily to prevent his tubes falling into the cavity. To this end he inserted a tube at one intercostal space, and bringing it out at another, he tied the ends together.

An accident called my attention to the fact that very powerful suction can be produced by very simple means. Desiring to have a flange on the outer end of the tube to prevent it from slipping completely into the cavity, I took a rubber tube and split the extremity for three-quarters of an inch. Putting the split end through the center of a disc of gutta-percha, I turned the two ends over on the disc and secured them by cementing another perforated disc of hot gutta-percha upon the top, thus holding the two halves of the rubber tube cemented between two discs of gutta-percha. After it was cool, I made traction on the tube, and found it so firmly attached that I considered it safe. Making an incision, and placing the tube in the patient's chest, I evacuated a large quantity of pus, the tube extending into the cavity, and the disc resting against the skin. At the close of the evacuation the air began to enter the tube at each inspiration. To prevent this, I placed my finger on the orifice of the disc during inspirations, but removed it during expirations, to allow the pus and air within to be expelled. In a few moments there was a strong suction on my finger at each inspiration, and lifting it, I was horrified to find the rubber tube being pulled from its hold between the gutta-percha discs. I pulled the disc away and saw the end of the tube slip out of sight under the skin. I plunged my finger after it, and just succeeded in pressing the rubber so firmly against the edge of a rib as to stop its progress, and then hooking a tenaculum into the rubber, through skin and all, I held it until it could be seized and extracted by forceps.

This accident showed how easy it is to produce a considerable vacuum in the chest without any complex apparatus; but it also showed the need of a better tube. To meet this necessity, E. H.

Sargent & Co., 125 State street, caused to be manufactured a quantity of tubes like that shown in the figure. The tube and flange are all one piece of soft rubber, strong enough to resist any suction they will ever meet.

This tube can be made to produce and maintain all the vacuum required. To this end a small conical cork should be cut to fit the orifice in the flange. After syringing out the chest with carbolized water, of the strength of one part to forty, sixty or eighty, whichever the surgeon may find sufficient to disinfect the case in hand, the patient is directed to draw in a full breath. He must then "hold his breath"—that is, close his larynx, and "press" or "bear down"; that is, make a strong expulsory effort with the larynx closed. This will drive out a large part of the air, and the remainder of the carbolized injection from the cavity. Now, pressing the finger on the orifice, let the patient draw in a new breath, and again removing the finger, repeat the expulsion. One or two forced efforts will make a pretty strong vacuum; or, if preferred, the surgeon can get up one nearly equal to it without any co-operation of the patient, by continuing for ten or fifteen breaths to apply the finger during inspiration and remove it during expiration. When the vacuum is sufficient, the cork should be inserted, and antiseptic dressings placed over all. The soft rubber flange acts as an air-tight valve against the skin, and the cork controls the orifice, so that no air can enter, and the vacuum will still be found exerting its power at the next dressing.

I think no effort should be made to obtain a very strong suction, lest it induce a possible hæmorrhage.

My experience in drainage tubes and carbolized injections for suppurating pleural cavities has been very favorable. Patients exhausted with hectic and suppuration return rapidly to health and strength, and go about their business, fat and ruddy, long before it is time to take away the tube, even when no suction is attempted. Exact statistics do not exist, within my knowledge, to show the amount of success of the vacuum plan, but, arguing on general principles, the rapid expansion of the lung could not be otherwise than beneficial. It will surprise me if so simple a device as the flanged soft rubber tube has not been already used; but, in a hasty search among authorities, I have not been able to find it mentioned, except in the valved or stop-cock form. However, the question of priority is of trifling consequence. The important point is to show the profession that with this simple tube, and a bit of cork, they can effect a continued suction upon the collapsed lung, and rapidly bring it out to the walls of the thorax. The same tube is useful in other large abscesses of various kinds. I placed one, not long since, in an abscess of the lung itself, which contained over half a pint of horribly offensive pus. The result has been surprisingly beneficial, the patient coming back almost from the gates of death under the use of daily antiseptic injections. However, the vacuum plan is not needed in this case, perhaps, and if so, an ordinary tube would have answered the purpose; but in cases where the pus occupies the pleura and collapses the lung, the faithful use of the vacuum tends to expand that organ and to prevent the formation of those chronic caverns, which sometimes require the resection of ribs for their care.

*No. 6 Sixteenth St., Chicago.*

## Figures and Tables

**Figure f1:**